# Hepatitis B virus infection and its determinants among HIV positive pregnant women: Multicenter unmatched case-control study

**DOI:** 10.1371/journal.pone.0251084

**Published:** 2021-04-30

**Authors:** Zelalem Alamrew Anteneh, Estifanose Wondaye, Endalkachew Worku Mengesha

**Affiliations:** 1 Department of Epidemiology and Biostatistics, School of Public Health, Bahir Dar University, Bahir Dar, Ethiopia; 2 Department of Infectious Disease Prevention & Control, Wagimera Zone Health Office, East Amhara Region, Ethiopia; 3 Department of Reproductive Health, School of Public Health, Bahir Dar University, Bahir Dar, Ethiopia; IRCCS Neuromed, CUAMM, ITALY

## Abstract

**Background:**

Hepatitis B virus (HBV) kills millions of people globally; it is worse in pregnant women. HBV and Human Immune Virus (HIV) co-infection is associated with increased liver diseases such as cirrhosis and hepatocellular carcinoma. This study aimed at identifying the determinants of HBV infection among HIV-positive pregnant women.

**Methods:**

A multicentre unmatched case-control study was conducted among 109 cases (HBV/HIV co-infected) and 327 controls (HIV positive) pregnant women in seven hospitals of the Eastern Amhara region. Interview and chart review data collection techniques were employed by trained personnel. A binary logistic regression model was used to identify independent predictors of hepatitis B virus infection. Variables with a p-value of <0.05 and 95% confidence interval for odds ratio not containing 1 considered independent predictors of HBV infection.

**Results:**

The findings of this study revealed that history of STI [AOR, 1.97, 95%CI, 1.09–3.56], hospital admission [AOR, 3.08, 95%CI, 1.69–5.61], traditional delivery care [AOR, 3.31, 95%CI, 1.72–6.37], family history of HBV [AOR, 3.33, 95%CI, 1.72–6.37], presence of opportunistic infections [AOR, 0.23, 95%CI, 0.12–0.58], viral load [AOR, 7.58, 95%CI, 3.18–8.01], CD4 count [AOR, 2.15, 95% CI, 1.01–4.59], anaemia [AOR, 3.07, 95% CI, 1.71–5.51] and unsafe sex [AOR, 1.98, 95%CI, 1.09–3.61] had a statistically significant association with HBV infection.

**Conclusions:**

Several exposure variables had statistically significant association with HBV infection. High Viral Load appeared to be the largest predictor of HBV infection in HIV patients. Therefore, targeted interventions such as behavioral change intervention for unsafe sex and STI should be in place, and screening tests and treatment at the early stage of conception for both partners is necessary.

## Introduction

Hepatitis B Virus infection (HBV) is a chronic inflammatory disease of the liver characterized by hepatocellular necrosis [1]. HBV is small, circular with a partially double-stranded genome of only 3200 bases. It is the smallest known human DNA virus presented with 5-types (A-E). The most abundant are non-infectious particles, containing HBsAg that measure 17 to 25 nm in diameter and are composed of lipid particles [2]. HBV infection is the causes of chronic hepatitis (CHB) worldwide, andone of the leading causes of morbidity and mortality regardless of the availability of an effective vaccine and the development of effective antiviral treatments [3].

Globally, nearly 240 million people are chronically infected with HBV, and approximately, 600,000 people die each year with HBV-related liver diseases. The highest prevalence was seen principally in Asia, sub-Saharan Africa, and the Pacific Islands [2]. Infection with HBV during pregnancy is associated with a high rate of vertical transmission (VT) and maternal mortality. Nearly, 85% of VT occurs in the peripartum period by the assimilation of infected maternal fluid, and the remaining 15% of VT occurs transplacentally. Pieces of evidence showed that 10%-20% of neonates born to mothers with hepatitis B surface antigen (HBsAg), and 90% of neonates born to mothers with both HBsAg and hepatitis B e antigen (HBeAg) more likely to be positive for HBV [4]. There is evidence showing HBV and HIV co-infection increases the risks of mortality among people livening with HIV. HBV infection can complicate the treatment of HIV patients on antiretroviral therapy resulting toxicity [5]. The co-infection with HBV can cause the HIV infection to evolve into AIDS, then to death; the progression is about two times more likely to occur among patients with co-infection compared to those with HIV infection alone [6].

Several factors can contribute to the occurrence of HBV infections and associated complications. These include delayed implementation of effective programs for screening and vaccination for new-borns [7], the lack of access to effective antiviral therapies, and early childhood horizontal transmissions [8]. HBV and HIV have shared the same route of transmission. They can be transmitted through exposure to semen, infectious blood, and other body fluids, from infected mothers to infants’, unsafe sexual intercourse, transfusions, presence of tattoos, injections, sharing of needles and syringes, and being health workers have been implicated as drivers of infection & transmission of HBV & HIV [9, 10]. Infection with HIV increases rates of HBV chronicity, prolongs the time the HBV stays in circulation and increases liver-related morbidity [11]. Evidence showed in people with HBV-HIV co-infection, HBV advances faster to cirrhosis, end-stage liver disease, and liver cancer than in people with only HBV infection [12]. Therefore, this study aimed to identify determinants of the presence of HBV/HIV infection among HIV-positive pregnant women.

## Methods and materials

A multicenter unmatched case-control study design was used to identify determinants of Hepatitis B virus infection among HIV-positive pregnant women from February-1 to April-31/2020 in the Eastern part of the Amhara region, Ethiopia. This study recruited seven hospitals out of the total eighteen hospitals in the region. A total of 436 (103 cases and 327 controls) were recruited for the study. We calculated the sample size using the unmatched case-control formula in EPI info. The following assumptions were used: confidence level 95%, power of 80%, case to control ratio 1:3, the proportion of exposure (history of STI) among controls, and cases is 9.8% and 26.2% respectively based on a previous study conducted in Lagos Nigeria [13]. Finally, we added 5% non-response rate to the calculated sample size.

Subjects were recruited from the registration books obtained from health facilities included in the study. The registration book is composed of lists of women with HBV/HIV co-infection, we recruited study participants consecutively till the calculated sample size was obtained.

### The population of the study

#### Cases (Co-infected HBV/HIV)

HIV-positive pregnant women with positive immune chromatographic confirmed Hepatitis B Virus infection.

#### Controls (None-HBV)

HIV positive pregnant women with negative immune chromatographic confirmed Hepatitis B Virus.

### Study variables

#### The outcomes of the study

Presence of HBV infection.

#### Independent variables

**Socio-demographic information:** age of mother, residence, maternal education, marital status, and maternal occupation.

**Knowledge of Mother:** about HBV transmission, control, and prevention. A mother is classified as knowledgeable if she is correctly answers greater than or equal to 70% of knowledge-related questions, otherwise not knowledgeable.

**Health-related factors:** history of IV drug use, symptoms of sexually transmitted infection (STI), blood transfusion, history of surgery, sooth extraction, hospital admission, history of abortion, place of delivery, participation in home delivery, caesarean section (CS) and immunization.

**Sexually transmitted infection (STI)**—was assessed as whether a woman had genital discharge and/or ulcer.

**Behavioral and cultural related characteristics:** multiple sexual partners, unprotected sexual intercourse, history of contact with HBV infected person, and history of the tattoo.

**HIV related factors:** viral load, CD4 cell count, WHO staging, opportunistic infections, duration of ARV treatment and treatment regimen.

### Data collection and data quality management

The questionnaire was prepared in English and translated to the local language Amharic, and back-translated to English to ensure validity. A data collection checklist was developed to capture the secondary data from hospital logbooks. We recruited six BSc and one diploma Prevention of Mother-To-Child Transmission (PMTCT) trained Midwives from PMTCT clinics of selected hospitals for data collection and two public health professionals were selected for supervision and follow-up of the data collection. The training was given to data collectors and supervisors on the method of interviews and how to extract the pertinent data by reviewing the patient’s records and how to fill the information on a structured questionnaire. Detailed training was given on how to handle and approach the patients and keeping the confidentiality of their information. A pre-test was done among 18 samples before the actual data collection to make corrections on the tool, and possible amendments were done to our questionnaire.

### Data management and analysis techniques

The data were coded and entered using Epi-Data version 3.1 and exported to SPSS version 23 for analysis. Descriptive statistics and chi-square tests were done to show baseline characteristics and the presence or absence of associations between predictors and HBV respectively. To identify factors associated with the presence of HBV among HIV-positive pregnant women; both bivariate and multivariate logistic regression analyses were performed. Each independent factor that could have plausible relations with HBV was entered into a bivariate logistic regression analysis to select candidate factors for the multivariate modeling. To be liberal, we used a p-value of 0.25 as a cut of point; all variables with a p≤0.25 in the bivariate regression model were selected for the multivariate logistic regression analysis. Variables with P value less than 0.05 in the final model were considered statistically significant predictors of HBV. Hosmer and Lemeshow p-value test was used to check model fitness. Multi-collinearity tests were performed to check the presence of correlations among independent factors. We computed the variance inflation factor (VIF) for each predictor variable by doing a linear regression of each predictor on all the other predictors; in each case, we obtained VIF within the range of recommended cut of points.

### Ethical clearance

Ethical clearance was obtained from Bahir Dar University ethical review committee, an official permission paper was obtained from Amhara Public Health Institute (APHI). Before the commencement of data collection, the purpose of the study was clearly explained and verbal informed consent was obtained from each study participant. A written consent can’t be administered in the country side of Ethiopia because a lot of people are unable to read and write. The Research and Ethical review committees in Ethiopia including the ethical review committee of Bahir Dar University approves an oral consent form. In this study, nearly 17% of the total study participants are unable to read and write. In this study participants were informed that they had the right to quit or withdraw from the study if they wanted to do. The privacy of our study participants is kept confidential by excluding personal identifiers from questionnaires.

## Results

### Sociodemographic characteristics of respondents

A total of 436 HIV-positive women (109 with HBV and 327 without) were interviewed in East Amhara hospitals. The Mean age of the respondents was 30(+/-) 5.33 and 30 (+/-) 6.08 years for cases and controls respectively. Nearly, three-quarters of both the cases and controls 84(77.06%) cases and 255(77.98%) respectively were urban residents. The majority of the cases 76(69.70%) and controls 246(73.90%) were orthodox religious followers. The majority of the study participants 75(68.8%) cases and 250(76.5%) controls were married. Nearly, one-fifth of 21(19.3%) cases and 64(19.6%) of controls had attended college and above educational level. Half 55(50.5%) of cases and 168(51.4%) of controls were unemployed (Table 1).

**Table 1:** Socio-demographic characteristics of HIV positive women at PMTCT in the hospital of Eastern Amhara, Ethiopia 2020.

**Factors**	**Cases (n = 109) N (%)**	**Controls (n = 327) N (%)**	**X**^**2**^	**p-value**
**Age in years**			6.8	0.15
**< = 24**	11(10.1)	63(19.3)		
**25–29**	40(36.7)	96(29.4)		
**30–34**	27(24.8)	84(25.7)		
**35–39**	26(23.9)	63(19.3)		
**> = 40**	5(4.6)	21(6.4)		
**Residence**			0.040	0.84
**Urban**	84(77.06)	255(77.98)		
**Rural**	25(22.94)	72(22.02)		
**Religion**			3.46	0.18
**Orthodox**	76(69.70)	246(75.20)		
**Muslim**	26(23.90)	72(22.00)		
**Protestant**	7(6.40)	9(2.80)		
**Ethnicity**			7.41	0.06
**Amhara**	79(72.5)	261(79.8)		
**Agew**	15(13.8)	37(11.3)		
**Tigray**	7(6.4)	22(6.7)		
**Oromo**	8(7.33)	7(2.1)		
**Marital Status**			4.2	0.12
**Married**	75(68.8)	250(76.5)		
**Single**	24(22.0)	45(13.8)		
**Divorced**	9(9.2)	32(9.8)		
**Educational Status**			4.2	0.39
**Unable to read & write**	21(19.3)	46(14.1)		
**Read And Write**	33(30.3)	101(30.9)		
**Primary School**	23(21.1)	61(18.7)		
**Secondary**	11(10.1)	55(16.8)		
**College and above**	21(19.3)	64(19.6)		
**Occupation status**			0.03	0.87
**Employed**	54(49.5)	159(48.6)		
**Unemployed**	55(50.5)	168(51.4)		

### Behavioral and health care factors for HBV infection among HIV positive pregnant women

Nearly one-third, 33(30.3%) of cases and 62(19.0%) of controls reported more than one sexual partner in the last one year. A majority, 76 (69.7%) of cases and 265(81.0%) of controls had gum tattooing practice. Nineteen (17.4%) of cases and 52(15.9%) of controls had tattoos on their body. About 58.7% of cases and 48.3% of controls had a history of IV drug use. Our data indicated that 59(54.1%) of cases and 100(30.6%) controls had a history of STI. The majority, 76(69.7%) of cases and 134(41.0%) of controls had a history of hospital admission. Two-fifths of 44(40.4%) cases and 52(15.9%) of controls had a history of participation in the activities of traditional birth care provision at home, who her self-assisted someone to give birth at home traditionally. One-third 42(38.5%) cases and 49(15.0%) controls had a family history of HBV infection (Table 2).

**Table 2:** Behavioural & health care factors for HBV infection among HIV positive women at PMTCT in Eastern Amhara, Ethiopia 2020.

**Factors**	**Cases (n = 109) N (%)**	**Controls (n = 327) N (%)**	**X**^**2**^	**p-value**
**Multiple sexual partners**			6.14	0.013
**Yes**	33(30.3)	62(19.0)		
**No**	76(69.7)	265(81.0)		
**Tattoo on the body**			0.14	0.708
**Yes**	19(17.4)	52(15.9)		
**No**	90(82.6)	275(84.1)		
**Gum tattooing**			0.23	0.633
**Yes**	76(69.7)	265(81.0)		
**No**	33(30.3)	62(19.0)		
**History of IV drug use**			3.54	0.06
**Yes**	64(58.7)	158(48.3)		
**No**	45(41.3)	169(51.7)		
**History of STI**			19.6	0.00
**Yes**	59(54.1)	100(30.6)		
**No**	50(45.9)	227(69.4)		
**History of blood transfusion**			0.074	0.79
**Yes**	12(11.0)	33(10.1)		
**No**	97(89.0)	294(89.9)		
**History of surgical procedure**			0.52	0.473
**Yes**	13(11.9)	48(14.7)		
**No**	96(88.1)	279(85.3)		
**History of tooth extraction**			0.11	0.74
**Yes**	26(23.9)	73(22.3)		
**No**	83(76.1)	254(77.7)		
**History of hospital admission**			27.06	0.000
**Yes**	76(69.7)	134(41.0)		
**No**	33(30.3)	193(59.0)		
**History of abortion**			0.79	0.372
**Yes**	15(13.8)	57(17.4)		
**No**	94(86.2)	270(82.6)		
**Traditional delivery care**			28.5	0.000
**Yes**	44(40.4)	52(15.9)		
**No**	65(59.6)	275(84.1)		
**Family with HBV**			27.45	0.000
**Yes**	42(38.5)	49(15.0)		
**No**	67(61.5)	278(85.0)		

### Clinical factors of HIV positive pregnant women

Nearly, 67.0% and 64.2% of the cases had baseline and current HIV viral load counts less than or equal to 1000. Similarly, 257(78.6%) and 296(90.5%) controls reported baseline and current viral load less than or equal to 1000 respectively. On the other hand, 33(30.3%) of cases and 160(48.9%) of controls had greater or equal to 500 cells/μl baseline CD4 count whereas 22(22.9%) of cases and 152(12.8%) controls reported greater or equal to 500 cells/μl current CD4 counts.

Three quarters 86(78.9%) of cases and 298(91.10%) control more than 10 months duration on ART. In addition, 48(44.0%) of cases and 78(23.9%) controls were at an advanced stage of baseline WHO clinical staging while, only 43(39.4%) cases and 57(17.4%) of controls were on the current clinical stage. This study indicated that 37 (33.9%) of the cases and 65 (19.9%) of the controls were reported opportunistic infection during the time of diagnosis. The majority, 100 (91.7%) of cases and 296 (90.5%) controls were RH+ by their blood groups. During the baseline assessment in the first appearance, 55(50.5%) of the cases and 72(22%) of the controls were anemic, however, only 10(9.2%) and 33(7.1%) cases and controls were anemic currently (Table 3).

**Table 3:** Clinical related factors among HIV positive pregnant women at PMTCT in eastern Amhara, Ethiopia 2020.

**Factors**	**Cases (n = 109) N (%)**	**Controls (n = 327) N (%)**	**X**^**2**^	**p-value**
**Baseline viral load**			5.99	0.014
**< = 1000**	73(67.0)	257(78.6)		
**>1000**	36(33.0)	70(21.4)		
**Current viral load**			41.9	0.000
≤1000	70(64.2)	296(90.5)		
**>1000**	39(35.8)	31(9.5)		
**Baseline CD4 count**			14.7	0.002
**<200**	33(30.3)	66(20.2)		
**200–350**	27(24.8)	49(15.0)		
**351–499**	16(14.7)	52(15.9)		
**> = 500**	33(30.3)	160(48.9)		
**Current CD4 count**			33.56	0.000
**<200**	32(29.4)	96(29.4)		
**200–350**	30(27.5)	37(11.3)		
**351–499**	25(20.2)	42(46.5)		
**> = 500**	22(22.9)	152(12.8)		
**Duration in ART**			11.65	0.001
**<10 months**	23(21.1)	29(8.9)		
**>10 month**	86(78.9)	298(91.1)		
**Baseline WHO staging**			16.21	0.000
**Pre advanced stage**	61(56.0)	249(76.1)		
**Advanced stage**	48(44.0)	78(23.9)		
**Current WHO staging**			22.42	0.000
**Pre advanced stage**	66(60.6)	270(82.6)		
**Advanced stage**	43(39.4)	57(17.4)		
**Presence of OIs**			9.03	0.003
**Yes**	37(33.9)	65(19.9)		
**No**	72(66.1)	262(80.1)		
**Baseline OIs**			10.58	0.001
**Presence of infection**	35(32.1)	57(17.4)		
**No infection**	74(67.9)	270(82.6)		
**Current OIs**			6.11	0.013
**Presence of infection**	86(78.9)	289(88.4)		
**No infection**	23(21.1)	38(11.6)		
**RH factor**			0.15	0.702
**RH+**	100(91.7)	296(90.5)		
**RH-**	9(8.3)	31(9.5)		
**Baseline Hgb level**			32.03	0.000
**Normal**	54(49.5)	255(78.0)		
**With anaemia**	55(50.5)	72(22.0)		
**Current Hgb level**			0.54	0.464
**Normal**	99(90.8)	304(93.0)		
**With anemia**	10(9.2)	33(7.1)		

### Determinants of HBV among HIV positive pregnant women

Based on the bivariate regression analysis, history of symptoms of STI, history of hospital admission, history of participation at a traditional birth, family history of HBV, multiple sexual partners, sexual intercourse without a condom, baseline viral load, current CD4 count, duration in ART, WHO clinical staging, presence of OIS, and a baseline Haemoglobin level of anemia had a p-value of less than 0.25, and were entered into the final model (Table 4).

**Table 4:** Bivariate regression analysis of determinants of HBV among HIV positive pregnant women at PMTCT in Eastern Amhara, Ethiopia 2020.

**Variables**	**Cases (n = 109) N (%)**	**Controls (n = 327) N (%)**	**COR**	**(95% CI)**	**P-value**
**Symptoms of STI**					
**Yes**	59(54.1)	100(30.6)	2.68	[1.72–4.18]	0.06
**No**	50(45.9)	227(69.4)	1(ref)		
**History of hospital admission**					
**Yes**	76(69.7)	134(41.0)	3.32	[2.09–5.28]	0.000
**No**	33(30.3)	193(59.0)	1(ref)		
**History of participation at a traditional birth**					
**Yes**	44(40.4)	52(15.9)	3.58	[2.21–5.81]	0.000
**No**	65(59.6)	275(84.1)	1(ref)		
**Family history of HBV**					
**Yes**	42(38.5)	49(15.0)	3.56	[2.18–5.81]	0.000
**No**	67(61.5)	278(85.0)	1(ref)		
**Sexual intercourse without using a condom**					
**Yes**	69(63.3)	160(48.9)	1.80	[1.15–2.81]	0.010
**No**	40(36.7)	167(51.1)	1(ref)		
**Multiple sexual partners**					
**Yes**	33(30.3)	62(19.0)	1.86	[1.13–3.04]	0.014
**No**	76(69.7)	265(81.0)	1(ref)		
**Baseline viral load**					
**< = 1000**	73(67.0)	257(78.6)	1(ref)		
**>1000**	36(33.0)	70(21.4)	1.81	[1.12–2.92]	0.015
**Current CD4 count**					
**<200**	32(29.4)	96(29.4)	2.3	[1.26–4.20]	0.006
**200–350**	30(27.5)	37(11.3)	5.6	[2.90–10.8]	0.000
**351–499**	25(20.2)	42(46.5)	4.1	[2.11–8.01]	0.000
**> = 500**	22(22.9)	152(12.8)	1(ref)		
**Duration in ART**					
**<10 months**	23(21.1)	29(8.9)	2.75	[1.51–4.99]	0.001
**>10 month**	86(78.9)	298(91.1)	1(ref)		
**Baseline WHO staging**					
**Pre advanced stage**	61(56.0)	249(76.1)	1(ref)		
**Advanced stage**	48(44.0)	78(23.9)	2.51	[1.59–3.96]	0.000
**Presence of OIs**					
**Yes**	37(33.9)	65(19.9)	2.1	[1.28–3.35)	0.003
**No**	72(66.1)	262(80.1)	1(ref)		
**Baseline Hgb level**					
**Normal**	54(49.5)	255(78.0)	1(ref)		
**With anaemia**	55(50.5)	72(22.0)	3.6	[2.28–5.70]	0.000

### Multivariable logistic regression analysis between HBV infection and determinant factors

In the multivariate logistic regression analysis, the Hosmer and Lemeshow model test p-value was 0.49.

The findings of the final condensed model showed that the odds of having HBV among pregnant women with a history of STI was 1.97 times more likely compared to those with no history of STI [AOR, 1.97, 95%CI, 1.09–3.56]. Pregnant women with experience of hospital admissions had more than 3 times likely to have hepatitis B virus infection compared to those who didn’t report hospital admissions [AOR, 3.08, 95%CI, 1.69–5.61]. Women who were providing traditional child delivery care services in villages were at higher odds of having B virus infection as compared to those with no history of traditional birth care service [AOR, 3.31, 95%CI, 1.72–6.37]. The family history of HBV showed more than three times higher risk of having HBV in cases than their counter controls [AOR, 3.33, 95%CI, 1.72–6.37]. The risk of having HBV infection among pregnant women with a viral load of more than 1000 copies/ml was more than seven times more likely compared to those with less than or equal to 1000 copies/ml in cases than their counterpart of controls [AOR, 7.58, 95%CI, 3.18–8.01].

Pregnant women with a current CD4 count less than 200 cells/mm^3^ had more than two times likely to report HBV infection compared to those with greater than 200 cells/mm^3^ [AOR, 2.15, 95% CI, 1.01–4.59]. Our study also depicted that pregnant women who had opportunistic infection had a higher risk of having HBV infection compared to those with no opportunistic infection [AOR, 2.54, 95%CI, 1.13–5.69], Study participants who had anemia were more than three times higher risk of having HBV infection compared to those with no history of anemia [AOR, 3.07, 95% CI, 1.71–5.51]. Eventually, our study showed that the odds of having sexual intercourse with no condom outside sexual partner was about two times more likely among women with HBV infection (cases) compared to women with no infection (controls) [AOR, 1.98, 95%CI, 1.09–3.61] (Table 5).

**Table 5:** Multivariate logistic regression analysis between HBV infection and determinant factors among HIV positive pregnant women attending PMTCT in eastern Amhara, Ethiopia 2020.

**Variables**	**Cases (n = 109) N (%)**	**Controls (n = 327) N (%)**	**AOR**	**(95% CI)**	**P-value**
**History of STI**					
**Yes**	59(54.1)	100(30.6)	1.97	[1.09–3.56]	0.024
**No**	50(45.9)	227(69.4)	1(ref)		
**History of hospital admission**					
**Yes**	76(69.7)	134(41.0)	3.08	[1.69–5.61]	<0.001
**No**	33(30.3)	193(59.0)	1(ref)		
**Provision of traditional delivery care**					
**Yes**	44(40.4)	52(15.9)	3.31	[1.72–6.37]	<0.001
**No**	65(59.6)	275(84.1)	1(ref)		
**Family history of HBV**					
**Yes**	42(38.5)	49(15.0)	3.33	[1.69–6.54]	<0.001
**No**	67(61.5)	278(85.0)	1(ref)		
**Unsafe Sex**					
**Yes**	69(63.3)	160(48.9)	1.98	[1.09–3.61]	0.025
**No**	40(36.7)	167(51.1)	1		
**Baseline Viral load**					
**< = 1000**	70(64.2)	296(90.5)	1(ref)		
**>1000**	39(35.8)	31(9.5)	7.58	[3.18–8.01]	<0.001
Current CD_4_ cell count					
**<200**	32(29.4)	96(29.4)	2.15	[1.01–4.59]	0.048
**200–350**	30(27.5)	37(11.3)	4.61	[1.99–10.6]	<0.001
**351–499**	25(20.2)	42(46.5)	4.21	[1.86–9.57]	0.001
**> = 500**	22(22.9)	152(12.8)	1(ref)		
**Presence of opportunistic infection**					
**Yes**	37(33.9)	65(19.9)	2. 33	[1.56–4.73]	<0.001
**No**	72(66.1)	262(80.1)	1(ref)		
**Baseline Hgb level**					
**Normal**	54(49.5)	255(78.0)	1(ref)		
**With anemia**	55(50.5)	72(22.0)	3.07	[1.71–5.51]	<0.001

## Discussion

Hepatitis B virus and HIV infections are causing paramount health challenges, especially in pregnant women and neonates. Co-infections of the hepatitis B virus and HIV is a common phenomenon often leading to an increased risk of morbidity and mortality compared to HBV or HIV mono-infection [14]. Monitoring the prevalence of HIV and HBV infections during antenatal care will help to design and implement timely interventions aimed at preventing perinatal transmission [14, 15].

The globe is working to eliminate HBV infection via sustainable development goals (SDG-3), taking into account the severity and burden of the disease. In 2016, the Global Health Sector Strategy (GHSS) on viral hepatitis called for the elimination of viral hepatitis as a major public health threat by 2030 (i.e. 90% reduction in incidence and 65% in mortality) with targeting Hepatitis B birth-dose vaccine coverage ≥ 95%, Hepatitis B third-dose vaccine coverage ≥ 95%, HBsAg testing coverage of pregnant women ≥ 95% [16].

HBV infection is one of the major challenges to good health across the globe. However, low and middle-income countries are disproportionately affected by the disease [17]. A lot of factors combine to cause a challenge to control infectious diseases including HBV infections. These include insufficient health care infrastructure, shortage of trained healthcare workers, poor access to health care, and difficulty in receiving appropriate interventions [18, 19]. Ethiopia is one of the low-income countries that is affected by hepatitis B infection [20, 21]. This requires a careful investigation of predictors among a unique domain of populations like pregnant women and HIV-infected individuals that highly vulnerable to preventable diseases. Thus this study sought to investigate modifiable risk factors to control HBV infections.

In this study, the odds of acquiring Hepatitis B virus infection among pregnant women who had a history of sexually transmitted infections were about two times higher than their counterparts. This is supported by the evidence that sexually transmitted infection (STI) is one of causes of HBV infection in HIV-positive pregnant women intensify the viral infection. This is might be due to patients who had a discharge and ulcer in their genitalia is easily exposed to transmissible diseases. Similar studies conducted in Kilimanjaro Tanzania and Lagos Nigeria showed similar findings with the present study [22, 23].

HIV-positive pregnant women with experience of hospital admissions showed more than 3 times higher risks of acquiring hepatitis B virus infection compared to those who didn’t have a history of hospital admissions. This could be attributed to poor infection prevention and control activities performed during hospital admissions, and this, in turn, might lead to hospital-acquired infections [24, 25]. This finding is supported by studies conducted in Jimma, and Bale Robe, and Southwest Ethiopia [26–28].

This study also showed that women who were providing traditional child delivery care services in villages were at higher risks of having hepatitis B virus infection compared to women with no history of providing traditional birth care services. This is might be due to repeated use of unsterilized instruments during delivery and not using infection protective tools that can prevent transmission of infections between traditional birth attendees and women giving birth such as gloves during traditional delivery care. The result is in line with studies conducted in Atat hospital and the North Shoa zone of the southern and western parts of Ethiopia [29, 30]. A family history of HBV showed more than three times higher odds of HBV infections among the cases than their counter controls. This is due to the close contact of family members with the HBV-positive patient in the family. Often sharing personal items such as toothbrushes, razors with an infected person may occur, and indirect transmission via blood from needle sticks or other sharp instruments contaminated with HBsAg of an infected person can happen in a family. This is supported by similar other studies conducted in Ethiopia [4, 31–33].

Our study showed that the odds of sexual intercourse without a condom outside sexual partner was about two times more likely among women with HBV infection (cases) compared to women with no infection (controls). This finding can be explained that the hepatitis B virus is blood-born virus; blood, semen, and other body fluids are a common source of infection. Thus sexual contacts serve as a mode of transmission for the agent. Thus, sexually active women have a higher chance of getting the infection especially those who have a behavior of dating multiple sexual partners without a condom. Therefore, change in sexual practice and behavioral modification is an important component of reducing hepatitis B infection. This finding is also supported by several studies conducted across sub-Saharan Africa [4, 26, 27, 32–41].

The odds of HBV infection among pregnant women with HIV viral load of more than 1000 copies/ml was more than seven times more likely compared to those with less than or equal to 1000 copies/ml in cases than their counterparts of controls. This might be because higher HIV viral load increased the susceptibility of infection by other viruses including HBV infections. The study is supported by other similar studies conducted in southeastern Ethiopia, in Jos Nigeria, and Kinshasa, the democratic republic of Congo [27, 34, 42]. The odds of having hepatitis B virus infection among pregnant women with CD4 less than 200, from 200–350, and 351–499 cells/mm3 was much higher than women with CD4 greater than 500 cells/mm3. This association might be because, at a lowered CD4 count, re-emergence of HBV replication occurs due to spontaneous reverse seroconversion marked by the disappearance of anti-hepatitis B surface antibodies and reappearance of HBsAg [43]. Our finding is supported by a study conducted in Wolaita Sodo Southern Ethiopia and a study conducted in the united states where CD4 count showed a significant association with HBV infections [33, 44].

Opportunistic infections have been shown to have an effect on HBV infection among immune-compromised people [45]. Our study revealed that the risk of having an opportunistic infection is more than two times likely to occur among HBV-infected HIV-positive pregnant women (cases) compared to those without HBV infection (controls). This is supported by a well-established fact that immunity decrement often leads to opportunistic infection especially in ART patients. This finding is supported by a similar study conducted in Bale robe, southeast Ethiopia [27].

This study also revealed that pregnant women with HBV infection were more than threefold at risk of having anemia compared to HIV-positive pregnant women without HBV infection (control). This finding is in accordance with similar other study findings where pregnant women with HBV infection more often to low hemoglobin level in the blood as compared to women without hepatitis B infection [46, 47]. However, a study conducted in Uganda showed that the presence of HBV infection among pregnant women is not associated with hemoglobin level in the blood, this variation may be attributed to lower sample size in the given study to produce a reliable estimate compared to our study, and nutritional status of HIV positive women at PMTCT might affect nutritional anemia [48].

This study investigated factors affecting the presence of HBV infection in a very narrow, highly vulnerable, and needy domain of population (HIV positive pregnant women); this contributes to the body of knowledge on risk factors for local and national level intervention. Besides, identifying the risk factors of HBV infection is an important contribution to the efforts have been carried out to prevent vertical transmission of infections in Ethiopia. The evolvement of seven hospitals in this study has an added value to enhance the external validity of our findings due to the rare nature of HBV/HIV co-infection. However, the co-infection has a serious public health impact on the mother and a new-born. So, the findings of this study can have a significant contribution to prevent and control the transmission HBV/HIV co-infection in low-income settings.

Despite these strengths, findings should be interpreted in the light of some limitations. The temporal relationship between HBV and HIV can’t be established. Pregnant women were screened for both HIV and HBV at the same time during their antenatal visits. Therefore, very difficult to say HBV infection occurred after HIV does, or the other way round. Similarly, the temporal relationship between independent factors and HBV infection can’t be established because of the use of cross-sectional data collection techniques. In addition, some exposure variables such as the history of STI were collected based on the symptomatic approach, merely on the women’s self-report that could result in recall bias or varies according to illness perception of the mother. Nevertheless, these biases are non-differential, as they are independent of the characteristics of women (Case vs. Control). In addition, our study was conducted on women who presented themselves for ANC care in health institutions, the findings don’t apply to women who failed to receive care for their pregnancy.

## Conclusions

This study sought to compare potential exposure to HBV within the domain of HIV-positive pregnant women attending PMTCT clinics in Eastern hospitals of the Amhara region. Several exposure variables were deemed to have increased odds of having HBV infection. Factors such as history of STI, hospital admission, provision of traditional delivery care, family history of HBV, unsafe sex, viral load, CD4 count, opportunistic infections, and presence of anemia were statistically associated with HBV infection. Therefore, an intervention targeting these factors is warranted to control the transmission of HBV infection in this domain of population. Based on our findings, pregnant women with a history of STI and hospital admission related to their HIV status should receive careful attention, proper intervention, and conscious follow-up to prevent the reactivation of HBV antigen or prevent the new introduction of the agent. Similarly, HIV-positive women with higher viral load and lower CD4 count should get prior attention to the care provided to pregnant women to prevent new occurrences of diseases including HBV infections. We strongly advise behavioral change interventions targeting the prevention of unsafe sex (sex with no condom outside marital partner) and awareness creation of women to refrain from providing traditional delivery care without appropriate protective equipment. Furthermore, both mother and husband (sexual partner) should receive a screening test and treatment at an early stage of conception.

## Supporting information

S1 Data (SAV)

S1 Questionnaire (DOCX)
