# Supplementary material for: Hepatitis B virus infection and its determinants among HIV positive pregnant women: Multicenter unmatched case-control study
Source: PLoS One. 2021 Apr 30;16(4):e0251084. doi: 10.1371/journal.pone.0251084 (PMC8087076; doi:10.1371/journal.pone.0251084)
Supplement: S1 Questionnaire — (DOCX) [file pone.0251084.s002.docx]

**S1 Questionnaire.**

HBsAg test result …………………. Patient code No ………………….

**Part –I Sociodemographic characteristics**

| S.no | Question | | Choose | Skip |
| --- | --- | --- | --- | --- |
| Part 1. Socio Demographic characteristics | | | | |
| 101 | Age ( in years ) | | ----------- |  |
| 102 | Where did you live (Kebele)? | | 1.Urban  2. Rural |  |
| 103 | What is your ethnicity? | | 1. 1.Agew 2. 2.Amhara 3. 3.Tigre 4. 4.Oromo 5. 5.Others specify |  |
| 104 | What is your religion? | | 1.Muslim   1. 2.Orthodox 2. 3.Protestant 3. 4.Others specify |  |
| 105 | What is your marital status? | | 1.Married  2. Single  3. Divorced  4.Widowed  5. Others …………. |  |
| 106 | What is your current occupation? | | 1. Self-employed  2. Student  3. House wife  4. Farmer  5.Government employee  6. Other (specify)------- |  |
| 107 | What is your Educational status? | | 1. Can’t read and write  2. Read and write only  3. primary ( 1-8 )  4. Secondary ( 9-12 )  5. College and above |  |
| Part –II Knowledge about HBV transmission | | | | |
| 201 | Have you heard of a disease, caused by hepatitis B virus? | | 1. Yes 2. No |  |
| 202 | Can hepatitis B aﬀect liver? | | 1. Yes 2. No 3. Not sure |  |
| 203 | Can hepatitis B cause liver cancer? | | 1. Yes 2. No 3. Not sure |  |
| 204 | Are nausea, vomiting, and loss of appetite common symptoms of hepatitis B? | | 1. Yes 2. No 3. Not sure |  |
| 205 | Can hepatitis B aﬀect all age groups? | | 1. Yes 2. No 3. Not sure |  |
| 206 | There is no symptom of hepatitis B in some patients? | | 1. Yes 2. No 3. Not sure |  |
| 207 | Can hepatitis B transmitted through contaminated blood? | | 1. Yes 2. No 3. Not sure |  |
| 208 | Can hepatitis B transmitted by blades of ear or nose pierces? | | 1. Yes 2. No 3. Not sure |  |
| 209 | Can hepatitis B transmitted by unsafe sex? | | 1. Yes 2. No 3. Not sure |  |
| 210 | Can hepatitis B transmitted by mother to child? | | 1. Yes 2. No 3. Not sure |  |
| 211 | Is hepatitis B curable/treatable? | | 1. Yes 2. No 3. Not sure |  |
| 212 | Is vaccination available for hepatitis B? | | 1. Yes 2. No 3. Not sure |  |
| Part III : Health related factors | | | | |
| 301 | History of IV drug use? | | 1. Yes  2. No |  |
| 302 | Have you ever had discharge and/or ulcer from genitals? | | 1. Yes  2. No |  |
| 303 | Did you have a history of Blood transfusion? | | 1. Yes  2. No |  |
| 304 | History of Surgical procedure (skin graft, amputation, or any operation within the body)? | | 1. Yes  2. No |  |
| 305 | History of Tooth extraction? | | 1. Yes 2. No |  |
| 306 | History of Hospital admission? | | 1. Yes 2. No |  |
| 307 | History of Abortion? | | 1. Yes  2. No  3. Not applicable | If no skip to Q-309 |
| 308 | If yes in Q-307 which type | | 1. Unsafe abortion  2. Safe abortion |  |
| 309 | Where did you deliver your last baby in last 5 yrs.? | | 1. health facility  2. at home  3. Not adequate | If at home skip to Q-311 |
| 310 | If you deliver in Health facility, where did you deliver? | | 1. Hospital  2. Health center |  |
| 311 | Have you ever attend in traditional birth at home? | | 1. Yes  2. No |  |
| 312 | Did you have History of Caesarean Section (CS)? | | 1. Yes  2. No  3. Not applicable |  |
| Part IV: Behavioural related factors Associated with HBV Co-infection | | | | |
| 401 | Did you have more than 1 sexual Partner in the last 1 yr.? | | 1. Yes 2. No |  |
| 402 | Have you ever did Sexual intercourse without using condom outside your husband in the last 1 year? | | 1. Yes   2. No |  |
| 403 | Family History with HBV infection? | | 1. Yes 2. No |  |
| 404 | Do you have ever tattooing on your body? | | 1. Yes 2. No |  |
| 405 | Do you have ever tattooing on your gum? | | 1. Yes 2. No |  |
| Part –V HIV related factors ( Patient’s Chart reviewing ) | | | | |
|  | | |  |  |
| 501 | Is she ever had immunization against hepatitis B in her time? | | 1. Yes  2. No | If no skip to 503 |
| 502 | If yes, how many doses she had taken? | | 1. One dose  2. Two dose 3. Three dose | |
| 503 | If she had not vaccinated what was a reason | | 1. Not accessible  2. Not interested 3. Not have Rx | |
| 504 | Is a Viral load done? | | 1. Yes  2. No | If not done skip to 505 |
|  | Baseline VL | | -------- |  |
|  | Current VL | | --------- |  |
| 505 | Is CD4 Cell count done? | | 1. Yes  2. No | If not done skip to 506 |
|  | Baseline CD4 | | -------- |  |
|  | Current CD4 | | -------- |  |
| 506 | Is WHO Staging is done? | | 1. Yes  2. No | If not done skip to 507 |
|  | Base line stage | | 1. Stage I 3. Stage III  2. Stage II 4. Stage IV |  |
|  | Current stage | | 1. Stage I 3. Stage III  2. Stage II 4. Stage IV |  |
| 507 | Is an Opportunistic infection? | | 1. Yes  2. No | If not done skip to 508 |
|  | Base line OI | | ------- | |
|  | Current OI | | -------- | |
| 508 | Is Treatment regimen specified? | | 1. Yes 2. No | If not done skip to 509 |
|  | Base line Regimen | | -------- |  |
|  | Current Regimen | | -------- |  |
| 509 | Duration of ARV treatment in month | | ----------------------------- | |
| 510 | When the HBsAg was reactive | |  | |
| 511 | Have Laboratory tests been done? | | 1. Yes 2. No | If yes please list below |
|  |  |  | Base line | Current status |
|  | 1. |  |  |  |
|  | 2. |  |  |  |
|  | 3. |  |  |  |
|  | 4. |  |  |  |
|  | 5. |  |  |  |
